# Establishment of a fluorescence staining method for *Schistosoma japonicum* miracidia

**DOI:** 10.1038/s41598-020-73526-x

**Published:** 2020-10-07

**Authors:** Yuanxi Shen, Rongyi Ji, Man Yang, Jiaojiao Lin, Hairong Wang, Chuangang Zhu, Qinkun Xu

**Affiliations:** 1 Key Laboratory of Animal Parasitology, Ministry of Agricultμre of China, Shanghai Veterinary Research Institute, Chinese Academy of Agricultural Sciences, No. 518, Ziyue Road, Min Hang District, Shanghai, 200241 China; 2 Henry Fok College of Biology and Agriculture, Shaoguan University, No. 288, University Road, Zhenjiang District, Shaoguan City, Guangdong Province China; 3 Shandong Provincial Engineering Technology Research Center of Animal Disease Control and Prevention, Shandong Agricultural University, 61 Daizong Road, Tai’an City, 271018 Shandong Province China

**Keywords:** Biological techniques, Immunological techniques

## Abstract

Currently the diagnosis of schistosomiasis is mainly determined by observing the presence of eggs in host stool samples. Because of the overwhelming number of impurities in the stool, eggs are rarely observed. Therefore, the stool hatching method is used to observe the miracidia in the water. However, the miracidia of *Schistosoma japonicum* are small and difficult to detect, and missed detection is likely to occur when the infection level is low. In this study, recombinant streptococcal protein G-enhanced green fluorescent protein (rSPG-EGFP) was expressed, purified, and used as a fluorescence staining reagent for miracidia. A preliminary miracidium fluorescence staining method based on antigen and antibody bindingwas established by combining recombinant protein staining with the stool hatching method. The stool hatching method was used to collect the miracidia of *S. japonicum*, and *Schistosoma*-positive serum and the recombinant protein were mixed to assess the feasibility of fluorescence staining of miracidia. The miracidia of *S. japonicum* and *Schistosoma turkestanicum* were incubated with *S. japonicum*-positive serum and *S. turkestanicum*-positive serum, respectively, to identify miracidia species. When the fluorescence staining method was used to observe living miracidia, the miracidiawere labelled by the recombinant protein, and their motility status was not affected.

## Introduction

Schistosomiasis is a zoonotic parasitic disease, and liver fibrosis caused by schistosome infection may eventually lead to serious consequences in patients, such as secondary portal hypertension, causing patients to lose their capacity to work and ability to provide self-care, even leading to death^1,2^. The control and elimination of schistosomiasis is a long-term and arduous task, and we must further intensify the control of the source of infection and improve the diagnostic capacity^3,4^. Currently, the main method for diagnosing schistosomiasis is an aetiological diagnosis, which includes a faecal examination and stool hatching and uses the observation of schistosome eggs or miracidia as the basis for the diagnosis of schistosomiasis^5^. Compared with the faecal examination method, the stool hatching method is a rapid and sensitive detection method that plays an important role in the prevention and control of schistosomiasis. In the *National Surveillance Protocol of Schistosomiasis* (2011), the stool examination (hatching method with nylon mesh bags)wasfirst used as one of the standard methods for the aetiological examination at surveillance sites in China^6^. The principle of stool hatching is that mature *S. japonicum* eggs quickly hatch miracidia from the host stool that swim in water when exposed to an appropriate temperature and light. Identification is based on the swimming characteristics of miracidia in water. This method is not only an important method for the detection of human schistosomiasis but also an extremely important method for the detection of schistosomiasis in livestock. However, the stool hatching method is often unable to accurately determine miracidia due to operators’ insufficient understanding of miracidia; in particular, when the number of miracidia is limited, missed detection is more likely to occur^7^. In addition, a host may have a complex infection. Due to the abundance of trematode categories and the similar morphology of miracidia, a misjudgement readily occurs. Therefore, hatched miracidiamust be identified using a more effective method.

Both the cercariae membrane reaction^8,9^ and circumoval precipitin test (COPT)^10,11^ indicate that antigens are present on the surface of eggs and cercariae. When specific antibodies are present in the examined serum, the antibodies specifically bind to antigens on the surface of eggs and cercariae, forming specific precipitates around eggs or a distinct transparent membrane or mantle on the surface of miracidia. Therefore, the antigen present on the surface of miracidia can be used to artificially label miracidia with a fluorescent protein through antigen and antibody reactions, increasing the sensitivity of the detection of miracidia in the stool hatching method and facilitating identification.

Streptococcal protein G (SPG) is a streptococcal cell wall protein that binds to a variety of human and animal IgG antibodies^12^. The C domain (containing domains C1, C2, and C3) of SPG at the COOH-terminus has been noted to affect the binding of SPG to the IgG Fc region. While the C1 and C2 domains differ in only two amino acids, the C1 and C3 domains have six amino acid inconsistencies. The IgG-binding capacity of the C3 domain is seven times higher than the C1 domain^13^. Therefore, in the present study, a fusion protein consisting of the IgG-binding domains of SPG (C3 domain)and enhanced green fluorescent protein (EGFP) was constructed. rSPG-EGFP retained both activities: the IgG-binding capability of SPG and fluorescence activity of EGFP. Using this recombinant protein, specific schistosome antibodies are bound and labelled at the surface of miracidia. The aim of this study was to improve the sensitivity of miracidium observationsin the stool hatching test, reduce the methodological difficulties encountered by researchers, improve the accuracy of diagnosis, and reduce the rate of missed detection.

## Results

### Construction, transformation, and characterization of the recombinant plasmid

**Figure 1:**
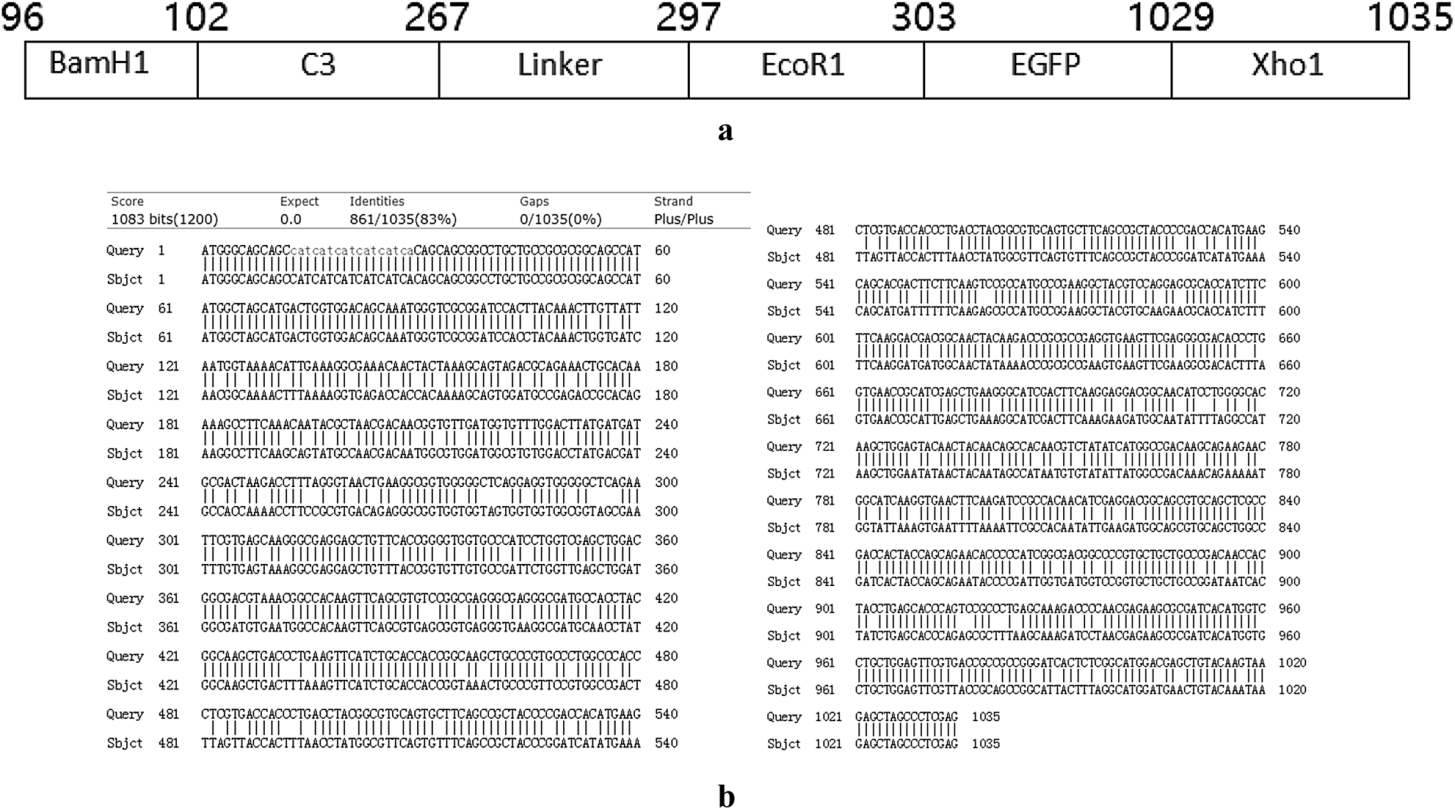
Sequence of the reconstructed rSPG-EGFP region. (**a**) Diagram of the C3-linker-EGFP sequence. (**b**) Comparison of the complete sequence of the recombinant protein rSPG-EGFP before and after codon optimization. Query: C3-EGFP sequence before codon optimization, Subject: C3-EGFP sequence after codon optimization.

The codon-optimized C3linkersequence and EGFP sequence were separately cloned into the pET28a ( +) plasmid to construct the recombinant pET28a ( +)-C3-linker and pET28a ( +)-EGFP plasmids. After sequencing validation, the EGFP sequence was amplified from PET-28a ( +)-EGFP, and the amplified EGFP sequence and pET28a ( +)-C3-linker plasmid underwent double digestion and ligation to construct the recombinant pET28a ( +)-C3-linker-EGFP plasmid. A diagram of the new gene produced after recombination is provided in Fig. 1a; a comparison of the recombinant protein sequences before and after codon optimization is shown in Fig. 1b.

**Figure 2:**
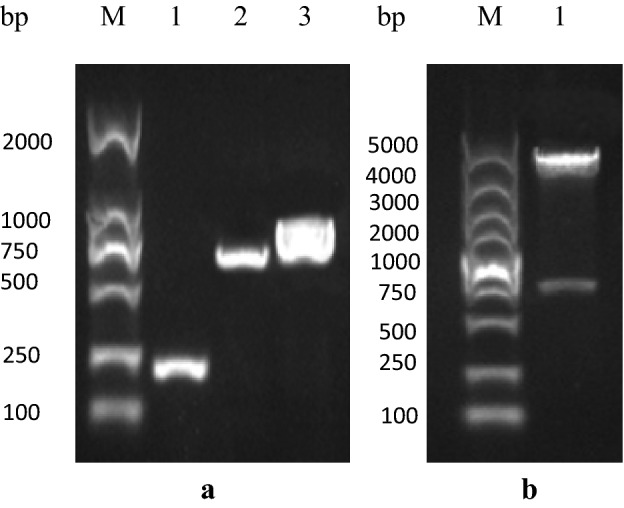
PCR and double digestion validation of the recombinant plasmid. (**a**) PCR validation of the recombinant plasmid: M: protein marker, 1: C3 sequence amplified by PCR with a size of 208 bp, 2: EGFP sequence amplified by PCR with a size of 724 bp,and 3: the complete C3-EGFP sequence amplified by PCR with a size of approximately 938 bp. (**b**) Double digestion validation of the recombinant plasmid: M: protein marker and 1: BamHI and XhoI double digestion to obtain the C3-EGFP fragment with a size of approximately 938 bp.

The reconstructed prokaryotic expression plasmid pET28a ( +)-rSPG-EGFP was characterized by polymerase chain reaction (PCR) and double digestion (Fig. 2). The full-length fragment of the target gene rSPG-EGFP was 939 bp, the C3linker fragment was 207 bp, and the EGFP fragment was 732 bp. The PCR and digestion results were consistent with the expected sizes of the fragments, and the sequencing result was consistent with the designed sequence.

### Expression and purification of the recombinant protein

**Figure 3:**
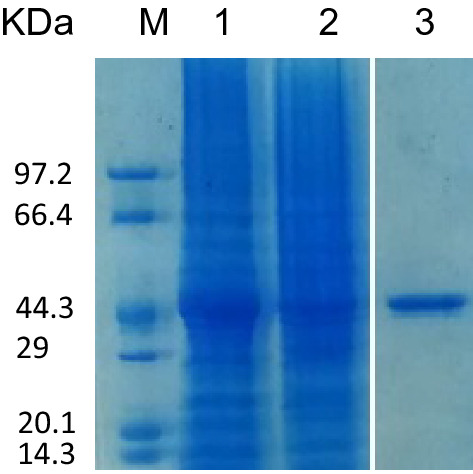
Solubility analysis of the recombinant protein. M: protein marker, 1: precipitate, 2: supernatant, and 3: purified protein.

The recombinant plasmid was transformed into *Escherichia coli* BL21(DE3) for the induced expression and purification of the recombinant protein. The sodium dodecyl sulfate–polyacrylamide gel electrophoresis (SDS-PAGE) solubility analysis showed that the recombinant protein rSPG-EGFP expressed from the recombinant plasmid pET28a ( +)-rSPG-EGFP in the *E. coli* BL21(DE3) host strain was present in both the ultrasonic supernatant and precipitate. After sonication, the protein in the supernatant was purified with Ni^2+^-nitrilotriacetic acid (Ni–NTA) resin, and a single protein band was obtained, as shown in Fig. 3. The molecular weight of the recombinant protein was approximately 44 kDa, consistent with the theoretical molecular weight.

### Identification of the binding activity of the recombinant protein rSPG-EGFP to IgG

**Figure 4:**
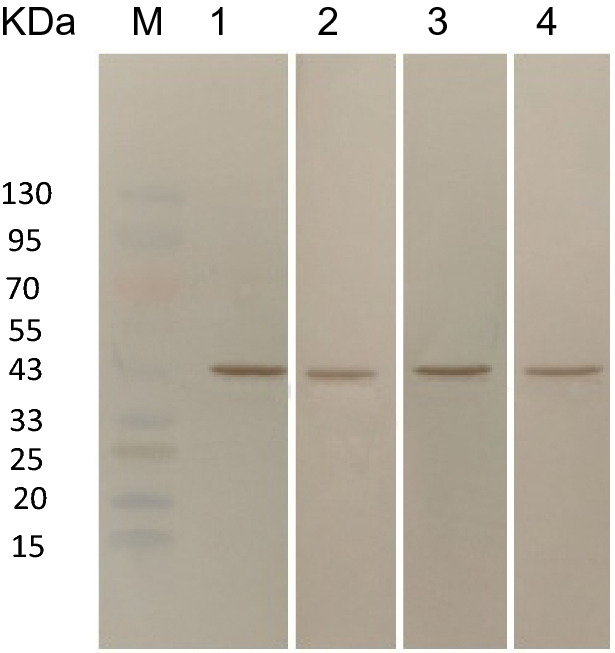
Qualitative characterization of the binding of the recombinant protein rSPG-EGFP to rabbit, bovine, mouse, and goat IgGsusing WB. M: protein marker, 1: binding of the recombinant protein to bovine enzyme-linked IgG, 2: binding of the recombinant protein to rabbit enzyme-linked IgG, 3: binding activity of the recombinant protein to goat enzyme-linked IgG,and 4: binding activity of the recombinant protein to mouse enzyme-linked IgG.

Western blotting (WB) and enzyme-linked immunosorbent assays (ELISAs) were used to qualitatively and quantitatively characterize the ability of the recombinant protein to bind rabbit, bovine, mouse, and goat IgGs. The recombinant protein had the ability to bind to rabbit, bovine, mouse, and goat IgGs (Fig. 4), and the binding constants were 3.2 × 10^7^, 1.14 × 10^8^, 2.6 × 10^7^, and 1.0 × 10^8^ Ka, respectively (Table 1).

**Table 1:** Affinity constants for the recombinant protein rSPG-EGFP.

Species	Affinity constant (Ka)
Bovine	1.14 × 10^8^
Goat	1.0 × 10^8^
Mouse	2.6 × 10^7^
Rabbit	3.2 × 10^7^

### Analysis of the fluorescence activity and the fluorescence characteristics of the recombinant protein

The fluorescence activity of the recombinant protein rSPG-EGFP was characterized. Changes in fluorescence inside the bacteria during expression were monitored in real time with a fluorescence microscope (Supplementary Fig. S1 online). After the addition of the inducer isopropyl-β-D-thiogalactopyranoside (IPTG), green fluorescence gradually appeared inside the bacteria, and the fluorescence intensity increased over time after expression.

The excitation and emission spectra of the recombinant protein were detected using an FLS1000 steady-state and time-resolved fluorescence spectrometer. The detected maximum excitation wavelength was 488 nm. When excited at 488 nm, the maximum emission wavelength of the recombinant protein was 510 nm; the changes in the fluorescence intensity of the recombinant protein are shown in Supplementary Fig. S2 online. Using the same excitation and emission wavelengths, the fluorescence intensity of the recombinant protein rSPG-EGFP was much higher than the commercial EGFP.

### Establishment of a fluorescence staining method for miracidias

*S. japonicum* and *S. turkestanicum*miracidia were obtained using the stool hatching method, and the morphology and motion trajectory of miracidia in water were observed macroscopically. *S. japonicum* miracidia and *S. turkestanicum*miracidia were visualized as macroscopic white dots that moved continuously in water, and the motion trajectories were all linear. In addition, both *S. japonicum* miracidia and *S. turkestanicum*miracidia moved upward and were phototropic. *S. japonicum* miracidiaaccumulated in large numbers on the surface of the water, while *S. turkestanicum*miracidiawere scattered in the water, swam fast, and were difficult to distinguish from moving *S. japonicum* miracidia.

**Figure 5:**
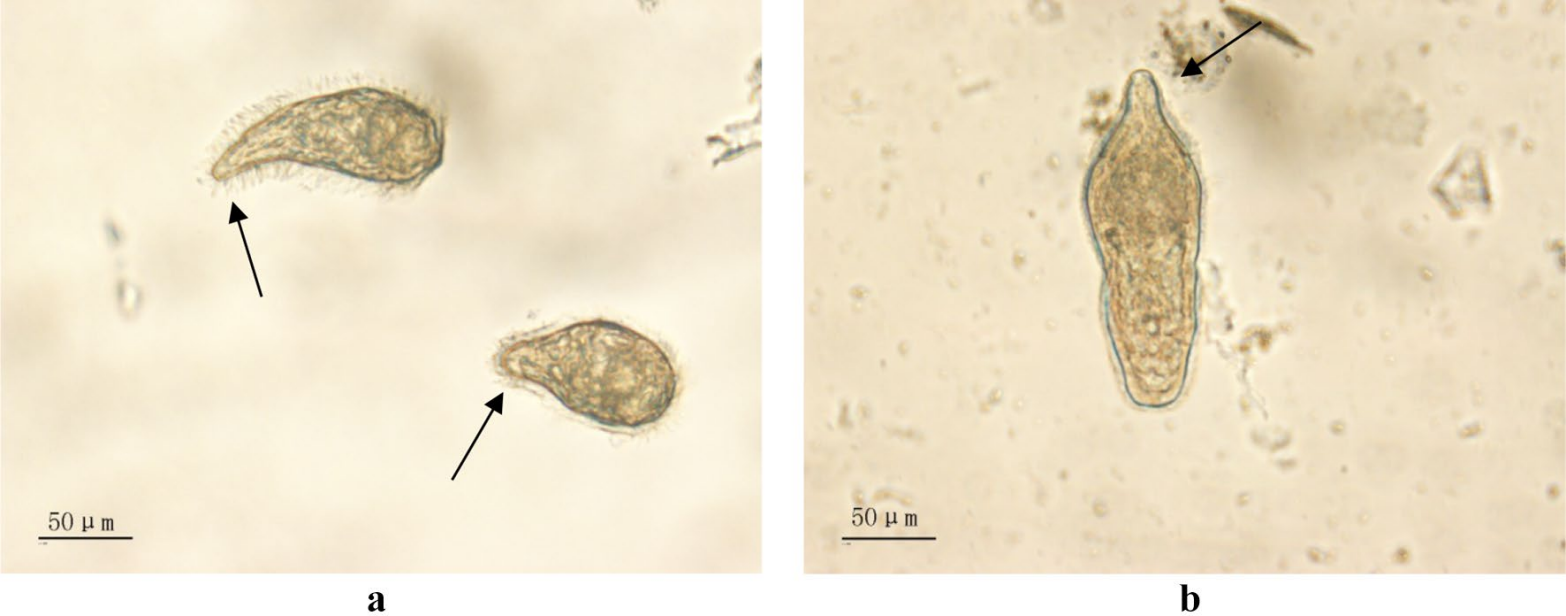
Microscopic observations of *S. japonicum* miracidia and *S. turkestanicum* miracidia. (**a**) *S. japonicum*miracidia; (**b**) *S. turkestanicum*miracidia; The arrows indicate the position of the apical gland in the miracidia.

After fixation, the shape of *S. japonicum* miracidia was extremely irregular (pear-shaped, ping-pong racket-shaped, melon seed-shaped, etc.) with left–right symmetry and an average size of 75–110 × 30–50 μm. However, *S. turkestanicum*miracidia were oval, pear-shaped, ping-pong racket-shaped, and melon seed-shaped, with cilia on the surface and an average size of 130–160 × 50–70 μm, which was larger than *S. japonicum* miracidia (Fig. 5). Both *S. japonicum*miracidia and *S. turkestanicum*miracidiaextend apical processes that are located at the anterior end of the body as a mouth-like protrusion. An apical gland is located in the centre of the anterior part of the body, which is observed as a pouch-like structure. Two lateral glands or head glands are located on the lateral sides slightly behind the apical gland in miracidia with a long pear shape, and both have openings at the apical processes. A square experiment was designed to explore the serum concentration and the recombinant protein concentration required for incubation; the results are shown in Table 2 (the corresponding images are presented in Supplementary Fig. S3 online). When different dilutions of serum and different concentrations of the recombinant protein were incubated with themiracidia, particularly noticeable differences in the amount of fluorescence were not observed under the fluorescence microscope. In summary, the most suitable serum dilution and concentration of the recombinant protein rSPG-EGFP for fluorescence staining of miracidia were set to 1:100 and 0.0025 mg/ml, respectively.

**Table 2:** Exploration of the optimal serum dilution and protein concentration for incubation.

Serum dilution	Protein concentration		
	0.01 mg/ml	0.005 mg/ml	0.0025 mg/ml
1:10	+ +	+ +	+ +
1:20	+ +	+ +	+ +
1:40	+ +	+ + +	+ + +
1:100	+ + +	+ + +	+ + +

“ + ” fluorescence intensity is weak; “ +  + ” fluorescence intensity is obvious; “ +  +  + ” fluorescence intensity is very strong.

**Figure 6:**
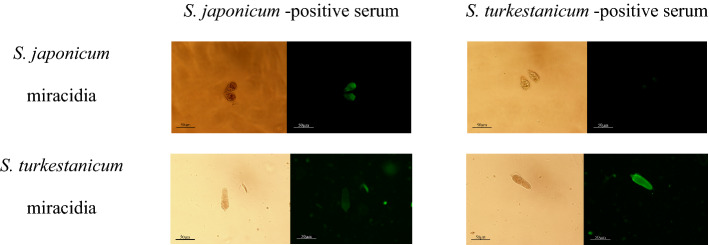
Analysis of the feasibility of using different schistosome-positive sera for the identification of miracidia species (20 ×).

Cross-serum experiments were designed based on the optimal serum dilution and the concentration of the recombinant protein rSPG-EGFP used in the incubation, as determined using the square experiment. As shown in Fig. 6, *S. japonicum* miracidia or *S. turkestanicum*miracidia were incubated with *S. japonicum-*positive serum or *S. turkestanicum-*positive serum, respectively; the remaining procedures were the same as described above. The results are shown in Fig. 6. When *S. japonicum* miracidia were incubated with the with *S. japonicum*-positive serum, they emitted fluorescence under a fluorescence microscope, and the fluorescence was observed throughout the body of the miracidia, revealing the overall morphology of miracidia. However, when *S. japonicum* miracidia were incubated with *S. turkestanicum*-positive serum, they did not emit fluorescence with the same intensity under a fluorescence microscope, showing only weak fluorescence. When *S. turkestanicum*miracidia were incubated with *S. japonicum*-positive serum and *S. turkestanicum*-positive serum, the miracidia incubated with *S. turkestanicum*-positive serum exhibited fluorescence throughout their bodies. Thus,the recombinant protein rSPG-EGFP was only able to bind the surface of the miracidia when the miracidia were incubated with the positive serum for the corresponding schistosome. The identification of miracidiawas preliminarily achieved by adding positive sera from different parasites as primary antibodies and fluorescent molecules as secondary antibodies.

### Detection and observation of living miracidiawith fluorescence staining

*S. japonicum* miracidia were hatched using the stool hatching method and incubated with positive serum and the recombinant protein according to the optimal protocol, and the miracidia were observed under a fluorescence microscope (20× magnification). When more water was available, the miracidia swam freely. Under the microscope, the miracidia swam extremely fast, and the fluorescent protein was enriched on the surface of miracidia. The miracidiaresembled a meteor crossing the night sky, which was more apparent when observed with the naked eye. The droplet containing miracidia was allowed to incubate for 5 min until the water evaporated naturally. The miracidia clung to the surface of the glass slide and exhibited slow peristalsis. A large amount of fluorescent proteins accumulated on the surface of miracidia, with an intensity much greater than the fluorescently stained miracidia after fixation. The dynamic observation of fluorescence staining of the miracidiumis presented inSupplementary Fig. S4 online.

## Discussion

Schistosomiasis japonica is a disease caused by *S. japonicum*parasitizing humans or mammals and is a serious zoonotic parasitic disease^14^. This disease is widely distributed in the Yangtze River Basin and 12 southern provinces, cities, and autonomous regions in China, which are important agricultural production bases in China. The disease seriously damages people’s health and economic development^15^, and it is one of the important public health issues in China^16^. Currently, the detection of *S. japonicum* is mainly based on laboratory tests combined with an epidemiological history survey and clinical manifestations. Laboratory tests mainly rely on antibody detection and aetiological detection. Among these tests, antibody detection, such as ELISA^17,18^, indirect haemagglutination assay (IHA)^19,20^, and colloidal gold-based immunochromatography^21,22^, are widely used due to their advantages of simplicity and rapidity. However, because schistosome-specific antibodies can exist in the body for a long time, according to statistics, most detection systems still detect the presence of antibodies in patients within 1–3 years after treatment; therefore, a previous infection and current infection are unable to be distinguished, and efficacy assessments cannot be performed. Thus, antibody detection is often only used for screening. When the presence of schistosome antibodies is determined, aetiological tests are required to confirm the diagnosis.

The aetiological detection of schistosomes is based on egg detection^23^ and the detection of miracidia after stool hatching^24^. Due to the large amount of faeces produced by livestock and the presence of higher levels of fibre in the faeces, egg detection is not a suitable option. Currently, the main method for the aetiological detection of schistosomiasis in livestock in epidemic areas in China is stool hatching, which is widely used to detect schistosomes in livestock. Sample collection using this method is convenient, the detection cost is low, and the results are reliable. It is a routine detection method for the diagnosis of schistosomiasis in livestock and is also the detection method used in national assessments. However, some experience is required to detect, observe, and judge the hatched miracidia using this method. Generally, the observation time is not less than 3 min, and a magnifying glass is required to assist with observations when necessary. After miracidia are detected, their swimming must be observed. After fixation with povidone-iodine solution, the morphology of the miracidia must be observed under a microscope (40 × to 100 ×) for identification and judgement. The process is cumbersome and timeconsuming. Moreover, protozoa are often confused with miracidia. This process requires that the examiner has an extensive understanding of the swimming status and morphology of miracidia; therefore, the requirement for the examiner is quite high. If the examiner does not have sufficient knowledge of miracidia, he/she may not be able to make an accurate judgement, and missed detection may occur, particularly when the number of miracidia detected is low. In addition, the host can be infected by multiple miracidia species or become infected multiple times; in particular, when the host is infected with different trematode species and is simultaneously infected with several trematode species, misinterpretation of the results may occur due to the morphological similarity of trematode miracidia. Therefore, the detection and identification of hatched miracidiarequire further improvement, including improvements in the sensitivity and accuracy of detection. The cercariae membrane reaction^8,9^ and COPT^10,11^ have been used to detect schistosomiasis japonica, and a specific precipitate appears around the eggs and cercariae. Therefore, antigens may be present on the surface of miracidia. Based on this premise, we proposed to improve the efficiency of miracidia observations by adding specific antibodies and fluorescent molecules to amplify the antigen signal on the surface of miracidia. By adding specific antibodies against different parasites and fluorescent molecules, we tried to identify miracidia species and conducted corresponding experiments.^29^

In the present study, the recombinant expression plasmid C3-EGFP was constructed and, when induced, expressed a recombinant protein with IgG-binding activity and green fluorescence. The fluorescent labelling of miracidiawas achieved by adding this protein in combination with schistosome-specific antibodies. In this study, we used the specific interaction between the antigen and the antibody not only for the identification of miracidia but also for inducing miracidiato fluoresce by adding the recombinant protein as a signalling molecule. Under a fluorescence microscope, the surface of miracidia exhibited a bright green fluorescence signal.

Schistosomiasis turkestanica is caused by *S.turkestanicum*, which belongs to Platyhelminthes: Trematoda: Digenea: Schistosomatidae^25^. According to phylogenetic studies of the Schistosomatidae based onmorphological cladistic analyses, *S. japonicum* and *S. turkestanicum* reliably differ in only one character, namely, the number of testes^26^. Using *S. japonicum* miracidia and *S. turkestanicum* miracidia as samples, the miracidia were identified under a fluorescence microscope by adding specific positive serum and the recombinant protein rSPG-EGFP. In addition, because the recombinant protein rSPG-EGFP contained the C3 region of SPG, it retained the ability of SPG to bind to the IgGsfrom multiple species. When tested using an ELISA, the recombinant protein showed the ability to bind to rabbit, bovine, mouse, and goat IgGs, indicating that the recombinant protein rSPG-EGFP bound tightly to the specific antibodies in the sera of the species mentioned above. Thus, using the fluorescence staining method for miracidia with the recombinant protein rSPG-EGFP as a signalling molecule, a specific antibody can be used to select the serum from multiple species, without the need to select different fluorescent proteins according to different species, which substantially reduces the cost of the fluorescence staining method for miracidia and avoids the experimental inconveniences caused by the use of different fluorescent proteins as signalling molecules. In the present study, we also incubated the living *S. japonicum* miracidiaobtained after stool hatching with*S. japonicum*-positive serum and the recombinant protein. The fluorescence microscopy images showed that the surface of the miracidia emitted fluorescence signals and that the miracidia swam rapidly in water, with extremely high recognizability. Based on these results, fluorescence staining was useful for the identification of living miracidia.

The fluorescence staining method for miracidia established in this paper requires a fluorescence microscope for observation; therefore, some difficulties exist regarding its large-scale application. The equipment is expensive and it is not easy to use in the field. However, with developments in the mechanization and electronization of medical devices, the automation and electronization of aetiological diagnostic methods for schistosomes are inevitable trends for development. Yuan et al.^27^ and Pei-Cai et al.^28^ used a dynamic automatic identification device to replace humans in the observation of miracidia, as the human eye was replaced with an automatic identification device for the observation of miracidia and combined with a new amplification device to establish automatic dynamic identification of schistosome miracidia, which substantially improved the efficiency and accuracy of schistosome miracidia detection. If this identification system is combined with the stool hatching method and the fluorescence staining method described in the present study and if a dynamic identification device is used to identify the swimming status of the bright green fluorescence spots in a dark environment, detection may be even more accurate and rapid.

## Conclusions

A fluorescence staining method for miracidiausing the recombinant protein rSPG-EGFP as a signalling molecule was successfully established. With rSPG-EGFP, a specific antibody selects the serum from multiple species, without the need to select different fluorescent proteins for different species, which substantially reduces the cost of the fluorescence staining method for miracidia and avoids the experimental inconveniences caused by the use of different fluorescent proteins as signalling molecules.

## Methods

### Serum samples

All serum samples were stored in the laboratory.

Buffaloes were artificially infected with *S. japonicum*(Chinese strain), and positive faecal examinations (hatching method) were recorded. Goats were naturally infected with *S. turkestanicum* according to positive faecal examinations (hatching method) in NimuCounty, Tibet.

### Construction, transformation, expression, and purification of the recombinant proteinrSPG)-EGFP

The sequences for EGFP and the C region of SPG were analysed, and a linker sequence was designed to connect the C3 sequence and the EGFP sequence. Codon optimization was performed for thesesequences to facilitate expression in prokaryotic cells. The above complete C3linker and EGFP sequences were synthesized. According to the sequences of EGFP and the C region of SPG, two pairs of primers containing restriction sites (Table 3) were designed to amplify the target sequences, which were cloned into the pET28a plasmid to construct the pET28a-C3-linker plasmid and the pET28a-EGFP plasmid. After the sequences were confirmed by sequencing, the EGFP sequence was amplified from the plasmid containing EGFP. Double digestion was performed for both the amplified EGFP sequence and the pET28a-C3-linker plasmid, and the digested DNA was gel-purified. The digested EGFP sequence was ligated to the digested plasmid to construct the recombinant pET28a-C3-linker-EGFP plasmid, which was then transformed into the *E. coli* BL21(DE3). After the recombinant plasmid was verified by PCR and double digestion, the sequence was confirmed by sequencing.

**Table 3:** Primer sequences and restriction sites.

Primer name (restriction site)	Primer sequence (5′ to 3′)
C3-linker region upstream primer (BanH1)	CGC GGATCC ACCTACAAAC
C3-linker region downstream primer (EcoR1)	CCG GAATTC GCTACCG
EGFP region upstream primer (EcoR1)	CCG GAATTC GTGAGTAAAG
EGFP region downstream primer (Xho1)	GGC CTCGAG GGCTAGCTCTTATTTG

After verification, protein expression was induced with IPTG, and the expressed protein was purified. The recombinant protein was analysed using SDS-PAGE.

### Analysis of the IgG-binding activity of the recombinant protein

WB was used to qualitatively identify the binding ability of the recombinant protein rSPG-EGFP to rabbit, bovine, murine, and goat IgGs(Jackson, PA, USA).. Fifteen microliters of the purified His-tagged recombinant protein (rSPG-EGFP) were separated on an SDS-PAGE gel and transferred to a 0.45 μm Poly(vinylidene fluoride)(PVDF) transfer membrane(Millipore, Darmstadt, Germany), which was blocked with 5% non-fat milk diluted with PBST (PBS containing 0.05% Tween), separately incubated with rabbit, mouse, goat, and bovine enzyme-linked secondary antibody IgG(diluted 1:2000 with PBST), and developed with a 3, 3′-diaminobenzidine (DAB) chromogenic solution(Leagene, Beijing, China).

ELISAs were used to quantitatively detect the binding affinity constants of recombinant protein rSPG-EGFP with IgGs from different species. The recombinant protein was diluted in a gradient and used as the antigen to coat a 96-well plate. After the plate was blocked with 5% non-fat milk, the serially diluted secondary antibody was added to the plate and allowed to bind to the antigen. Tetramethylbenzidine (TMB) was used for colour development, and 2 M H_2_SO_4_ was used to stop colour development. The optical density at 450 nm (OD450) was used as the ordinate and the logarithm of the antigen concentrations was used as the abscissa to establish a standard curve. According to the standard curve, K = (n − 1)/n[Ab]t ' − [Ab]t was used to calculate the affinity constant Ka. The average Ka value of three tests was recorded as the affinity constant for the binding of the recombinant protein to IgG.

### Analysis of the fluorescence activity of the recombinant protein

The fluorescence activity of the recombinant protein in bacterial culture was observed using a fluorescence microscope. Samples were collected before and at 1, 2, 4, and 6 h after IPTG induction to observe the fluorescence in bacteria under a fluorescence microscope. The differences in the in vivo fluorescence of bacteria before, during, and after the induction of the expression of the recombinant protein were observed.

An FLS1000 steady-state transient fluorescence spectrometer was used to detect the fluorescence spectra of the recombinant protein. Commercial EGFP(Beyotime, Shanghai, China) was used as a control and PBS was the dilution media. The excitation spectra of the recombinant protein rSPG-EGFP and the control protein were obtained by scanning 0.01 mg/ml of the corresponding protein solution using the spectrometer, and the maximum excitation wavelength was identified. The maximum excitation wavelength was then used to excite the recombinant protein and the control protein to obtain the emission spectra. Changes in the fluorescence intensity of the recombinant protein rSPG-EGFP and commercial EGFP were compared.

### Establishment of the fluorescence staining method for miracidia

#### Hatching, collection, and observation of miracidia

Faecal samples from New Zealand white rabbits artificially infected with *S. japonicum* were collected, the stool hatching method^7^ was used to obtain hatched miracidia, and the morphology and the movement patterns of miracidia in water were observed. Miracidia were collected, fixed with formaldehyde, and stored at 4 °C until use. *S. turkestanicum* were collected and observed using the same method.

Microscopic observations of *S. japonicum* and *S. turkestanicum*miracidiawere conducted. Approximately 100 miracidia were aspirated and centrifuged at 10,000 rpm for 2 min. The supernatant was discarded, and the miracidia were resuspended in 200 µl of normal saline and centrifuged. The supernatant was discarded; the wash was repeated twice. The miracidia were resuspended in 100 µl of normal saline, and 20 µl of the suspension were observed under a microscope.

### Exploration of the optimal conditions for fluorescence staining

Approximately 200 fixed *S. japonicum* miracidia were aspirated and centrifuged at 10,000 rpm for 2 min, and the supernatant was discarded. *S. japonicum* miracidia were resuspended in 200 µl of normal saline and centrifuged, and the supernatant was discarded. The wash step was repeated twice. The miracidia were resuspended in 200 µl of normal saline for the combined staining array. *S. japonicum*-positive serum was added at four dilutions: 1:10, 1:20, 1:40, and 1:100. After thorough mixing, the mixture was incubated at 37 °C for 30 min. Then, the mixture was centrifuged at 5000 rpm for 2 min, and the supernatant was discarded. The precipitate was washed twice with normal saline, and the miracidia were resuspended in 200 µl of normal saline. The recombinant protein rSPG-EGFP was added to the suspension in a dropwise manner, and the titrated final concentrations of the recombinant protein were 0.01 mg/ml, 0.005 mg/ml, and 0.0025 mg/ml. The mixture was incubated at 37 °C for 30 min and centrifuged at 5000 rpm for 2 min. The supernatant was discarded, and the precipitate was washed twice with normal saline. The miracidia were resuspended in 50 µl of normal saline, and 20 µl were observed under a fluorescence microscope. Supplementary Fig S5 online shows the whole progress described above.

### Specificity of the fluorescence staining method for the detection of Schistosoma japonicum

According to the optimal serum dilution and the incubation concentration of recombinant protein rSPG-EGFP determined above, *S. turkestanicum* miracidia and *S. japonicum* miracidia were incubated with positive serum from the same or different species; the remaining procedures were the same as described above. The staining results were observed using a fluorescence microscope to determine the feasibility of using sera from animals infected with different parasitesfor the identification of miracidium species.

### Detection and observation of living miracidiausing fluorescence staining

The routine stool hatching method^7^ was used to hatch and collect *S. japonicum* miracidia. According to the optimal serum dilution and recombinant protein concentration determined above, positive serum and the recombinant protein were added, and the mixture was incubated at 37 °C for 30 min. Then, 50 µl were used to observe the staining and activity of miracidiaunder a fluorescence microscope.

### Ethical approval

This study have been approved by the Institute’s Ethical Committee of Shanghai Veterinary Research Institute, Chinese Academy of Agricultural Sciences. The ethical approval Number is SHVRI-SZ-20181016-01.

## Human and animal rights

All the procedures involving animals were conducted in strict accordance with the Regulations for the Administration of Affairs Concerning Experimental Animals (Date issued: 1988.11.1), and all efforts were made to minimize suffering.

## Informed consent

Data sharing is not applicable to this article, as no datasets were generated or analysed during the current study.

## Supplementary information

Supplementary Information 1.
